# 17 β-Estradiol Impedes Aortic Root Dilation and Rupture in Male Marfan Mice

**DOI:** 10.3390/ijms241713571

**Published:** 2023-09-01

**Authors:** Louis Saddic, Sean Escopete, Lior Zilberberg, Shannon Kalsow, Divya Gupta, Mansoureh Eghbali, Sarah Parker

**Affiliations:** 1 Department of Anesthesiology and Perioperative Medicine, David Geffen School of Medicine, University of California, Los Angeles, CA 90095, USAmeghbali@g.ucla.edu (M.E.); 2 Department of Cardiology, Smidt Heart Institute, Cedars-Sinai Medical Center, Los Angeles, CA 90048, USAlior.zilberberg@cshs.org (L.Z.); shannon.kalsow@gmail.com (S.K.); divya.gupta@cshs.org (D.G.)

**Keywords:** estrogen, aneurysm, Marfan, TNFα, NF-κB

## Abstract

Marfan syndrome causes a hereditary form of thoracic aortic aneurysms with worse outcomes in male compared to female patients. In this study, we examine the effects of 17 β-estradiol on aortic dilation and rupture in a Marfan mouse model. Marfan male mice were administered 17 β-estradiol, and the growth in the aortic root, along with the risk of aortic rupture, was measured. Transcriptomic profiling was used to identify enriched pathways from 17 β-estradiol treatments. Aortic smooth muscle cells were then treated with cytokines to validate functional mechanisms. We show that 17 β-estradiol decreased the size and rate of aortic root dilation and improved survival from rupture. The Marfan transcriptome was enriched in inflammatory genes, and the addition of 17 β-estradiol modulated a set of genes that function through TNFα mediated NF-κB signaling. In addition, 17 β-estradiol suppressed the induction of these TNFα induced genes in aortic smooth muscle cells in vitro in an NF-κB dependent manner, and 17 β-estradiol decreased the formation of adventitial inflammatory foci in aortic roots in vivo. In conclusion, 17 β-estradiol protects against the dilation and rupture of aortic roots in Marfan male mice through the inhibition of TNFα-NF-κB signaling.

## 1. Introduction

Thoracic aortic aneurysms (TAAs) are among the most dangerous types of cardiovascular disease due to the high morbidity and mortality associated with acute dissection [1]. Epidemiological studies have consistently demonstrated sex disparities among patients with TAAs, with a higher prevalence in men (1.7:1) [2,3]. Much of what we know about TAAs comes from work on abdominal aortic aneurysms (AAA), which have been more extensively studied and which share some structural and biological properties with TAAs. Similar sex disparities exist in AAAs, and animal models of this disease have shown that female sex protection may be driven by 17 β-estradiol as mice prone to AAA and treated with 17 β-estradiol have smaller abdominal aortic diameters while similar mice undergoing ovariectomy have the opposite effects [4,5]. Nevertheless, there are also many mechanistic differences between TAAs and AAAs, which in part is due to the fact that many forms of TAA are due to heritable gene mutations. One of these heritable forms of TAA is Marfan syndrome, which results from an autosomal dominant mutation in the fibrillin-1 *(FBN1)* gene. Patients with this disease develop severe aortic root dilation early in life, which carries a high dissection risk. FBN1 is a structural protein of the microfibril network component of the extracellular matrix. How mutations in FBN1 lead to aortic dilation is still not fully understood, as recent studies challenging the role of transforming growth factor β (TGFβ) have come to light [6], but the mechanism likely involves mechanical signaling from aortic wall stress. Even less is known about the causes of sex bias in disease severity seen in Marfan patients. For example, despite an equal probability of inheriting FBN1 mutations, males are more likely than females to have aortic root dilation and dissection [3,7]. Likewise, studies in the Marfan *Fbn1^C1041G/+^* mouse, a model of Marfan that mimics a known human mutation and which results in severe aortic root dilation, show that male *Fbn1^C1041G/+^* mice have larger aortic root diameters compared to females after correction for body size [3,7,8,9,10]. In this same model, inhibition of androgen receptors by flutamide treatment in male mice attenuates TAA growth in part through blocking TGFβ signaling [11]. Interestingly, it has been shown that flutamide treatment can enhance circulating 17 β-estradiol levels, which was not examined in prior work [12,13]. Despite a clear role for 17 β-estradiol in protection from AAA, parallel studies to examine the potential therapeutic effects of 17 β-estradiol in *Fbn1^C1041G/+^* mice or any other model of heritable TAAs have yet to be performed. A clear understanding of mechanisms conferring protection due to the female sex could contribute to therapeutic interventions for both males and females that will allay disease severity and risk for dissection.

The protective effects of 17 β-estradiol against vascular injury have been studied in many contexts, including hormonal regulation of extracellular remodeling and atherosclerosis [14]. Many of these therapeutic actions have been linked to 17 β-estradiol’s ability to curtail inflammation. Specifically, 17 β-estradiol has been shown to attenuate both the attraction of inflammatory cells and the upregulation of inflammatory markers following vascular injury [15]. Increased inflammation has been documented in degenerative forms of AAAs and TAAs in the presence of atherosclerosis [16]. Nevertheless, inflammation may also play a role in many hereditary and syndromic forms of TAAs affecting younger patients, including those with Marfan syndrome. Aortic tissue from Marfan patients has increased infiltration of macrophages, B cells, and CD4+ and CD8+ T cells [17]. In addition, fibrillin-1 fragments from mice may be able to attract macrophages directly [18].

In this study, we demonstrate the effects of 17 β-estradiol on the development and rupture of aortic aneurysms in the Marfan mouse model *Fbn1^C1041G/+^*. We provide evidence that 17 β-estradiol downregulates TNFα stimulated NF-κB pro-inflammatory genes in vivo and in smooth muscle cells in vitro. These findings provide insight into potential mechanisms for the observed sex differences in the prevalence and outcomes of patients with TAAs, including Marfan syndrome, and thus they could unmask novel therapeutic targets for both sexes.

## 2. Results

### 2.1. 17 β-Estradiol Attenuates Aortic Root Enlargement in Marfan Mice

**Figure 1:**
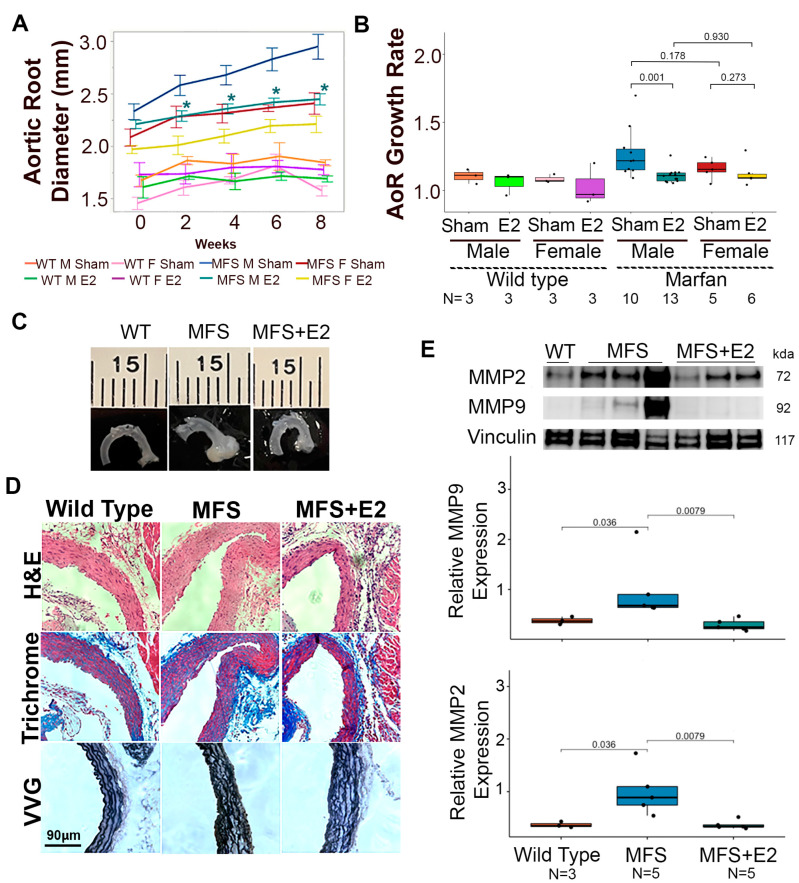
17 β-estradiol attenuates aneurysm growth in male Marfan mice. (**A**) Aortic roots measured every two weeks from 8-week-old wild-type male (*n* = 3), wild-type female (*n* = 3), wild-type male + 17 β-estradiol (*n* = 3), wild-type female + 17 β-estradiol (*n* = 3), Marfan male (*n* = 10), Marfan male + 17 β-estradiol (*n* = 13), Marfan female (*n* = 5), and Marfan female + 17 β-estradiol (*n* = 6) mice. Data are mean +/− standard error. * denotes *p* < 0.05 comparing Marfan male to Marfan male + 17 β-estradiol at each time point beyond 2 weeks. (**B**) The ratio of aortic root diameter at eight weeks over baseline. Data are median and interquartile range. Mouse cohorts as in (**A**). (**C**) Representative aortic images from male wild-type, Marfan, and Marfan + 17 β-estradiol mice. (**D**) Representative H + E, trichome, and elastin sections. Mouse cohorts as in (**C**). Scale bar = 90 μm. (**E**) Western blot with quantification of aortic root lysates from male wild-type (*n* = 3), Marfan (*n* = 5), and Marfan + 17 β-estradiol (*n* = 5) mice probed for matrix metalloproteinases and normalized to vinculin expression. E2: 17 β-estradiol, MFS: Marfan syndrome, Sham: sham surgery, WT: Wild-type.

Similar to previous studies examining the role of sex hormone regulation in TAA formation in the *Fbn1^C1041G/+^* mouse model [11], eight-week-old adult mice were treated in the presence or absence of 17 β-estradiol for eight weeks with serial imaging every two weeks via transthoracic echocardiography. Male Marfan mice treated with 17 β-estradiol for 4, 6, and 8 weeks had significantly smaller aortic root diameters compared to littermate male Marfan mice treated with sham surgery. The mean aortic diameter of 17 β-estradiol treated males tended to trend closely to the mean aortic diameter of littermate female Marfan mice, which was also significantly smaller than that of male Marfan mice. Female Marfan mice treated with 17 β-estradiol also tended to have decreased root diameters compared to littermate female Marfan mice, but this difference was not statistically significant (Figure 1A). Aortic root growth, as calculated by the quotient of aortic root diameter at 16 weeks of age over the aortic root diameter at baseline, was significantly higher in the Marfan male mice (median 1.22 IQR 1.1–1.4) compared to Marfan male mice treated with 17 β-estradiol (median 1.11 IQR 1.1–1.1) (*p*-value = 0.001). There was a trend towards higher aortic root growth in female Marfan mice (median 1.15 IQR 1.1–1.2) compared to those treated with 17 β-estradiol (median 1.09 IQR 1.1–1.2), but this was not statistically significant (*p*-value = 0.273) (Figure 1B). There were no significant differences in the change in ascending aorta diameter or weight throughout the eight-week study (Supplemental Figure S1B,C). Despite changes in root size on gross inspection (Figure 1C), Marfan male mice and littermates treated with 17 β-estradiol had intact intimal layers and similar degrees of elastin breaks (Figure 1D and Figure S1D). Treatment with 17 β-estradiol did, however, result in reduced levels of two well-established molecular markers of aneurysm pathology, matrix metalloproteinase-2 (Mmp2) and matrix metalloproteinase-9 (Mmp9), in male Marfan mice (Figure 1E).

### 2.2. 17 β-Estradiol Prolongs Survival in an Aortic Rupture Mouse Model

**Figure 2:**
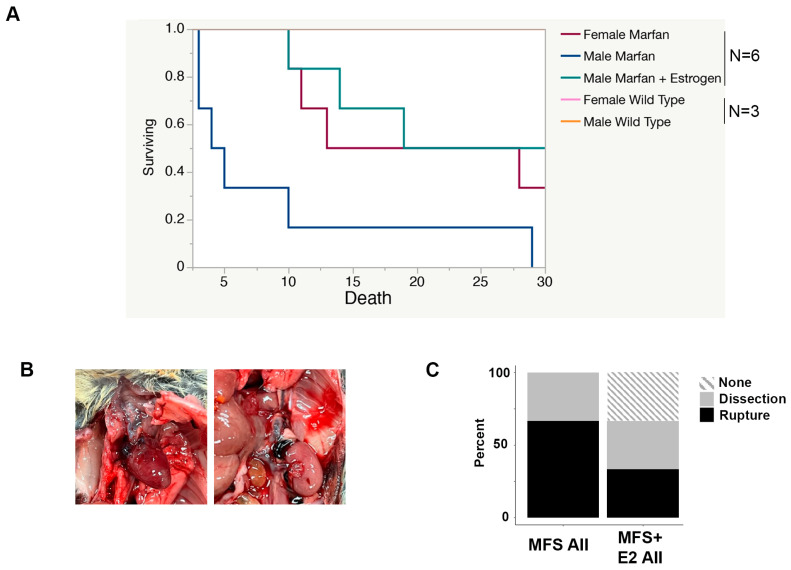
17 β-estradiol protects against aortic rupture in Marfan male mice treated with angiotensin II. (**A**) Survival curve of Marfan male, Marfan female, wild-type male, wild-type female, and Marfan male mice pretreated with four weeks of 17 β-estradiol following continuous infusion of angiotensin II. (**B**) Representative pictures of thoracic (**left**) and abdominal (**right**) aortic rupture in Marfan male mice treated with continuous infusion of angiotensin II. (**C**) Percentage of aortic pathologies found on gross and histological examination of aortas from angiotensin II treated Marfan males in the presence or absence of 17 β-estradiol. AII: angiotensin II, E2: 17 β-estradiol, MFS: Marfan syndrome.

Despite significant dilation, histological disorganization, and progressive growth in Marfan aortic roots, unchallenged *Fbn1^C1041G/+^* Marfan mice rarely dissect or rupture. Therefore, we employed the established approach of an angiotensin II challenge to provoke a more consistent phenotype severity, promote progression to dissection/rupture, and evaluate the potential beneficial effect of 17 β-estradiol [19]. We observed 100% death within 30 days in male Marfan mice treated with angiotensin II. Female Marfan mice treated with angiotensin II had a significantly higher rate of survival compared to males (*p* = 0.02), as did Marfan male mice treated with 17 β-estradiol (*p* = 0.01). Angiotensin II did not affect the survival of wild-type male or female mice, who exhibited 100% survival after 30 days of Angiotensin II infusion (Figure 2A). On gross examination, most Marfan mice died from aortic rupture in various locations, including the aortic root, the ascending aorta, the aortic arch, the descending thoracic aorta, and the abdominal aorta (Figure 2B). Those Marfan mice without evidence of rupture had signs of aortic root dissection on histological examination. Marfan mice treated with 17 β-estradiol had an equal percentage of dissected aortas but less rupture and more mice without evidence of rupture or dissection (Figure 2C).

### 2.3. 17 β-Estradiol Attenuates an Inflammatory Molecular Phenotype in Male Marfan Mice

**Figure 3:**
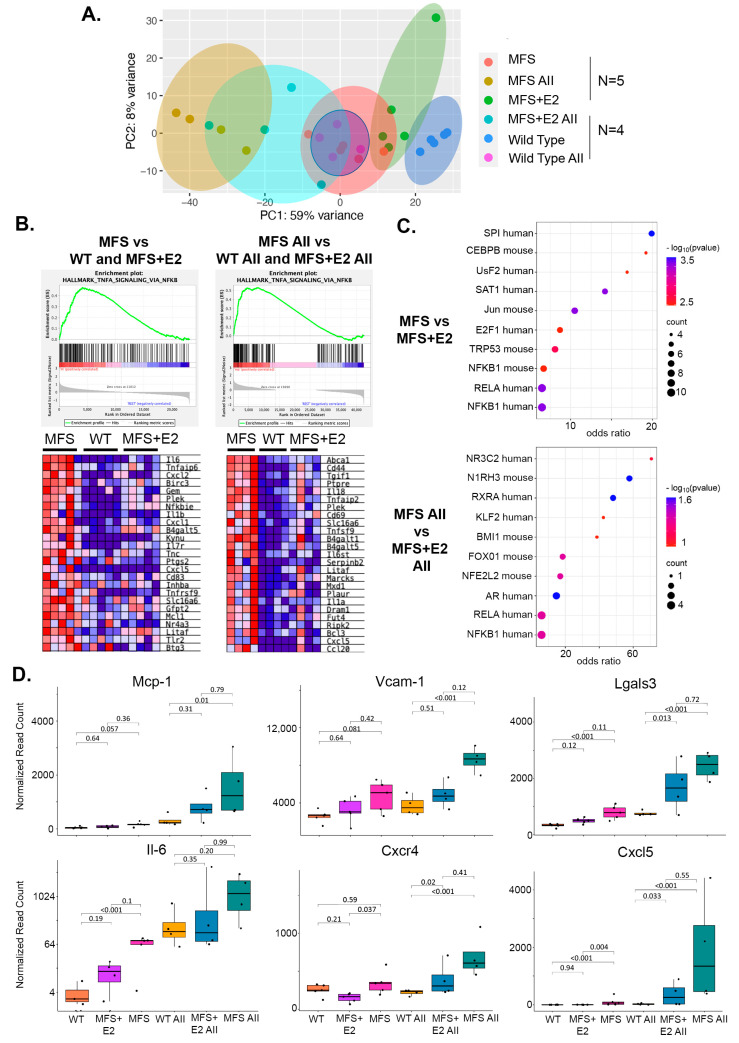
Transcriptomic profiling indicates that 17 β-estradiol blocks TNFα-NF-κB signaling and inflammation (**A**) Principle component analysis plot from RNAseq performed on aortic root tissue of male wild-type (*n* = 5), Marfan (*n* = 5), Marfan treated with eight weeks of 17 β-estradiol (*n* = 5), and seven days of angiotensin II treated wild-type (*n* = 4), Marfan (*n* = 4), and Marfan mice treated with five weeks of 17 β-estradiol (*n* = 4). (**B**) Gene enrichment analysis of the hallmark gene set TNFα signaling via NF-κB comparing Marfan mice to a cohort of combined wild-type and Marfan mice treated with 17 β-estradiol (**left**). The same analysis in angiotensin II treated mice (**right**). (**C**) Dot plot of transcription factors with targets enriched in Marfan mice compared to Marfan mice with 17 β-estradiol using the TRRUST database (**top**). The same analysis was in angiotensin II-treated mice (**bottom**). (**D**) Normalized read counts of *Mcp-1*, *Vcam-1*, *Lgasl3*, *Il-6*, *Cxcr4*, and *Cxcl5* gene expression from the same mouse cohorts outlined in (**A**). Box plots describe quartiles. Adjusted *p*-values from DESeq2 for pairwise comparisons are depicted in charts. AII: angiotensin II, E2: 17 β-estradiol, MFS: Marfan syndrome, WT: wild-type.

To gain insight into potential underlying mechanisms of 17 β-estradiol protection, we performed transcriptomic profiling on aortic roots from littermate 16-week-old male wild-type (*n* = 5), Marfan (*n* = 5), and Marfan mice treated with eight weeks of 17 β-estradiol (*n* = 5). We also profiled aortic roots from 13-week-old male wild-type (*n* = 4), Marfan (*n* = 4), and 17 β-estradiol treated Marfan mice (*n* = 4) exposed to continuous infusion of angiotensin II for seven days, a timepoint close to the inflection point of death in the Marfan mice and five days after the 48 h required to reach steady-state drug levels from the osmotic pumps employed. Principle component analysis demonstrated that global gene expression changes of Marfan aortic roots and wild-type aortic roots treated with angiotensin II diverge from wild-type tissue by a similar magnitude. This divergence is even more extreme in the Marfan male mice treated with angiotensin II. 17 β-estradiol pulls the global gene expression closer to the wild-type in both the angiotensin II treated and non-angiotensin II treated cohorts (Figure 3A). The activity of 17 β-estradiol remained functionally high throughout both the angiotensin and non-angiotensin groups, as indicated by the strong induction of the bona fide 17 β-estradiol target gene *Greb1* in the mice given 17 β-estradiol pellets compared to controls (Supplemental Figure S2A). Using gene set enrichment analysis (GSEA), we compared Marfan mice to a combined cohort of wild-type and 17 β-estradiol treated mice and observed significant enrichment in the hallmark gene set TNFα signaling via NF-κB (Figure 3B). A similar enrichment was noted when we compared Marfan mice with angiotensin II to a combined cohort of wild-type angiotensin II and 17 β-estradiol angiotensin II treated mice. In addition, differentially expressed genes upregulated in Marfan mice and suppressed with 17 β-estradiol in the presence and absence of angiotensin II were enriched in NF-κB target genes in the TRRUST database of human and mouse transcription factors (Figure 3C). Among the most differentially expressed of these NF-κB target genes are proteins known to play a role in phenotype switching of aortic smooth muscle cells (SMCs) to a more inflammatory-like state and include monocyte chemoattractant protein-1 (*Mcp-1)*, vascular cell adhesion molecule-1 (*Vcam-1)*, Galectin 3 (*Lgals3)*, interleukin-6 (*Il-6)*, CXC motif chemokine receptor 4 (*cxcr4)*, and CXC motif chemokine ligand 5 (*cxcl5)* (Figure 3D). A similar increase in TNFα expression was observed in Marfan mice compared to wild-type and Marfan mice with 17 β-estradiol (Supplemental Figure S2B). An opposite trend existed with SMC contractile markers such as α*SMA*, transgelin (*Tagln*), myosin heavy chain 11 (*Myh11*), and calponinin (*Cnn1*), which were downregulated in Marfan mice but to a lesser degree in those Marfan mice treated with 17 β-estradiol in the presence of angiotensin II (Supplemental Figure S2C).

### 2.4. 17 β-Estradiol Inhibits TNFα Mediated NF-κB Target Gene Expression in Marfan Aortic Smooth Muscle Cells

**Figure 4:**
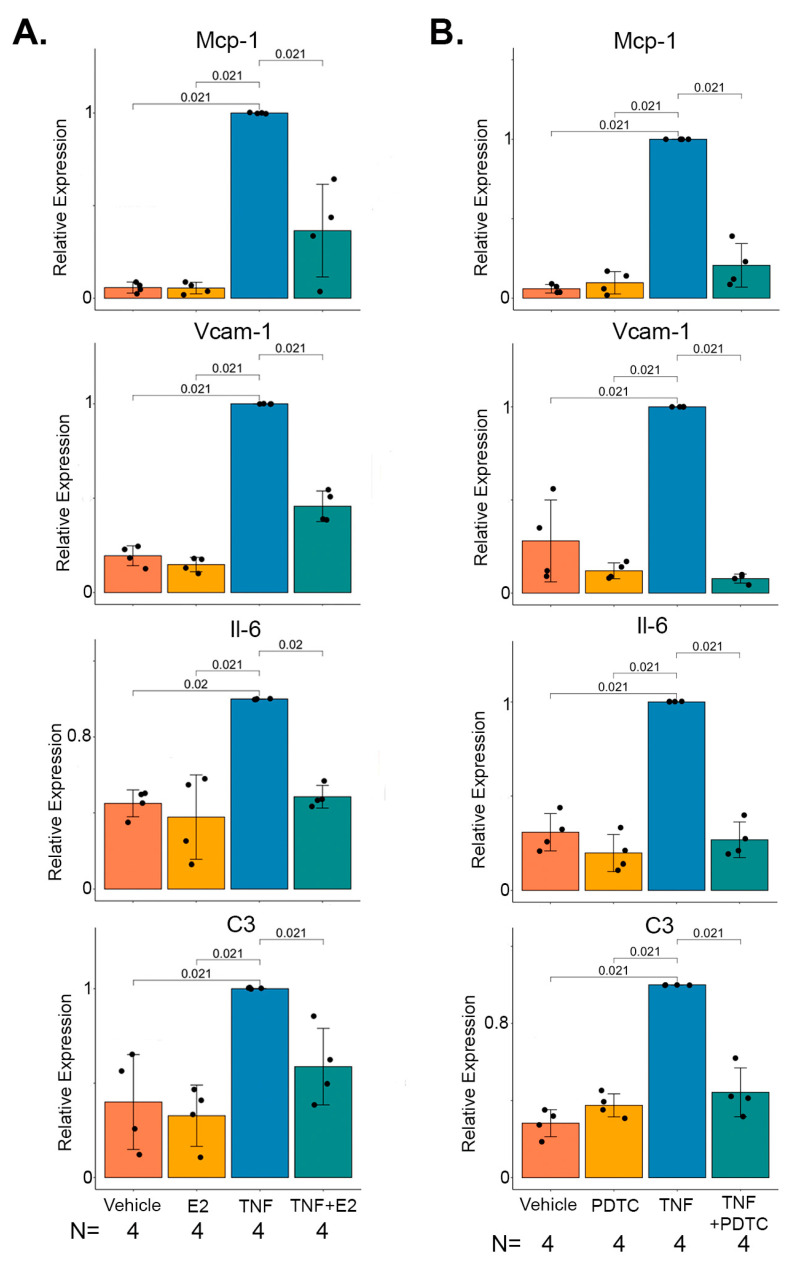
17 β-estradiol mitigates TNFα medicated NF-κB gene expression in murine aortic smooth muscle cells (SMCs). (**A**) Expression of *Mcp-1, Vcam-1, Il-6, and C3* in *n* = 4 independent Marfan male aortic SMC lines pre-treated with 17 β-estradiol or vehicle followed by the addition of TNFα or vehicle. (**B**) Expression of genes as in (**A**). following pre-treatment with PDTC or vehicle followed by the addition of TNFα or vehicle. Bar charts display mean +/− standard error. Relative expression is normalized to the cytokine or drug-alone treated group. E2: 17 β-estradiol, PDTC: Pyrrolidine Dithiocarbamate.

Previous studies have shown that 17 β-estradiol can block TNFα induced NF-κB target genes in many cell lines, including rat aortic SMCs [20]. To determine if the gene expression changes in NF-κB target genes uncovered in our bulk RNA-seq experiments could be taking place in SMCs and establish that 17 β-estradiol can directly modulate these effects, we performed in vitro experiments on aortic SMCs harvested from four independent male Marfan mice. Stimulation of these cells with TNFα induced the expression of the NF-κB target genes *Mcp-1*, *Vcam-1*, *Il-6*, and *C3*. Pre-treatment with 17 β-estradiol significantly inhibited the induction of these genes by TNFα (Figure 4A). Similar results were found when the same experiment was conducted on male human SMCs (Supplemental Figure S3). To demonstrate that 17 β-estradiol mediated these effects through NF-κB, aortic SMCs from Marfan mice were treated with TNFα in the presence or absence of the NF-κB inhibitor pyrrolidine dithiocarbamate (PDTC). As expected, PDTC blocked the TNFα mediated induction of *Mcp-1*, *Vcam-1*, *Il-6*, and *C3* (Figure 4B).

### 2.5. 17 β-Estradiol Prevents Inflammatory Foci in the Adventitia of Marfan Aortic Roots

**Figure 5:**
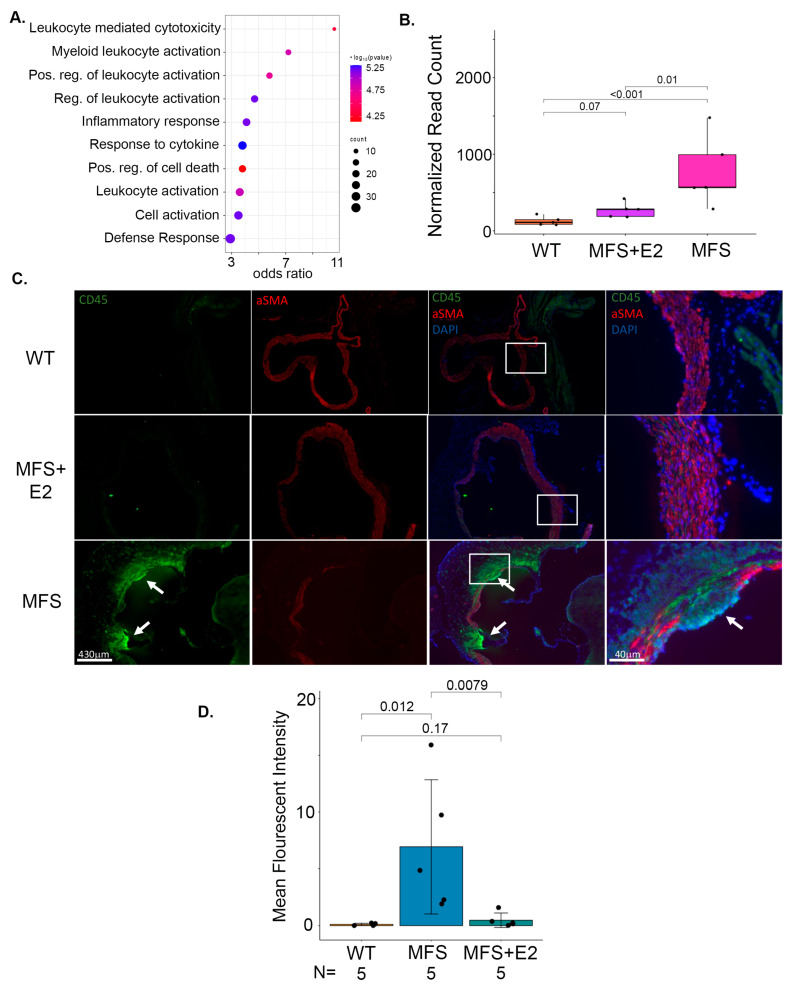
17 β-estradiol prevents inflammatory foci in the adventitia of Marfan mice. (**A**) Top enriched biological process ontology terms from the RNA-seq genes upregulated in Marfan compared to 17 β-estradiol treated Marfan male mice. (**B**) RNA-seq normalized read counts of *CD45* gene expression from male wild-type, Marfan, and 17 β-estradiol-treated Marfan mice. The box plot describes quartiles. Adjusted *p*-values from DESeq2 for pairwise comparisons are depicted above the chart. (**C**) Representative images of immunofluorescence staining of aortic roots from the same cohorts described in (**B**). Tissue is probed for CD45, αSMA, and DAPI nuclear stain. White arrows indicate CD45^+^ inflammatory foci. Scale bars are included below each panel. The far right panel is a higher magnification of overlay. (**D**) Mean fluorescent intensity of CD45^+^ stain from *n* = 5 mice in each cohort described in (**D**). *p*-values for pairwise comparisons are shown above the chart. E2: 17 β-estradiol, MFS: Marfan syndrome, WT: wild-type.

In general, adventitial infiltration of immune cells is a well-characterized component of aortic injury in Marfan mice. Similarly, in our transcriptomic analysis, gene ontology biological process enrichment of the upregulated genes in Marfan mice compared to Marfan mice with 17 β-estradiol mostly involved inflammatory terms (Figure 5A). CD45 was also among the top induced differentially expressed genes in the Marfan cohort compared to Marfan mice treated with 17 β-estradiol (Figure 5B). Since CD45 is a universal marker of immune cells, these findings suggest that 17 β-estradiol results in less inflammatory infiltration in the aortic wall of Marfan mice. To confirm this, we performed CD45 staining of aortic roots. There were clear foci of CD45-positive cells in the adventitia in the majority of tested Marfan mice, whereas no such foci were identified in any of the wild-type or Marfan treated with 17 β-estradiol mice. Quantification using the mean fluorescent intensity of CD45 around the aortic root showed a significant increase in staining in Marfan compared to wild type and Marfan with 17 β-estradiol mice (Figure 5C,D).

## 3. Discussion

In this study, we show for the first time that 17 β-estradiol treatment attenuates aortic root growth and reduces angiotensin II-mediated aortic rupture in a mouse model of hereditary TAA. These findings are consistent with prior evidence supporting 17 β-estradiol mediated protection in AAA [5,21], and extends this knowledge to the etiologically and mechanistically distinct yet to date unexamined hereditary TAA. Interestingly, female mice treated with an additional 17 β-estradiol also trended toward having a slower progression of aortic root growth compared to female mice treated with sham surgery, but this effect was substantially less prominent compared to males, not statistically significant, and did not fully return female Marfan root dimensions to wild-type levels. The reduction in aneurysm size correlated with reduced expression of matrix metalloproteinases Mmp2 and Mmp9. 17 β-estradiol has been shown to both directly regulate the expression of these extracellular matrix proteins and interact with several upstream molecular pathways that converge on the control of their expression [22]. Our data are consistent with prior investigations of male-biased TAA severity in Marfan syndrome. However, previous work has focused on androgen signaling [11]. Notably, these studies have shown that androgen receptor inhibition by flutamide attenuated aortic root growth, which at face value suggests that our results with 17 β-estradiol may complement or act via similar, directionally opposed mechanisms relative to androgens. Our observation that, while potently reducing aortic dimensions, 17 β-estradiol did not reverse or completely stop the growth of aortic roots through the 8-week time course is consistent with this possibility. Importantly, though, as mentioned above, androgen receptor inhibition has been shown to elevate circulating 17 β-estradiol levels [12,13], which was not examined in the previous study. This observation, coupled with our data, indicates that protection from aneurysm growth after flutamide treatment may be at least somewhat mediated by a direct 17 β-estradiol-related effect. Certainly, there remains significant room for additional studies to better understand how sex hormones impact bias in aneurysm and dissection severity in Marfan and other hereditary aortopathies.

Translational utility of the knowledge that 17 β-estradiol protects against aneurysm progression in males will rely upon a more defined molecular mechanism downstream of global hormone therapy, as estrogen supplementation to treat aneurysms would likely be an unpalatable option for most male patients. Therefore, we deployed transcriptomic profiling to clarify mechanisms downstream of 17 β-estradiol that could be leveraged for future therapies. These global gene expression changes demonstrated that 17 β-estradiol blocked the induction of a set of genes highly enriched in TNFα mediated NF-κB inflammatory signaling. Interestingly, many previous studies have already shown that 17 β-estradiol can block TNFα mediated NF-κB gene induction in many cell types, including SMCs, through several mechanisms, including the stability of IκBα, blocking NF-κB nuclear translocation, and the direct inhibition of gene expression on promoters [23,24,25]. We find this intriguing as recent single-cell RNA-seq experiments in the same Marfan animal model have identified a set of modified SMCs that express many NF-κB target genes, such as Vcam-1, that likely underwent a phenotypic switch from a contractile to a more inflammatory-like state [26]. Our in vitro experiments on cultured SMCs from Marfan mice are consistent with this hypothesis as 17 β-estradiol repressed the induction of similar pro-inflammatory genes by TNFα in an NF-κB dependent manner. There are many examples of this type of SMC phenotypic switch in vascular disease mediated by TNFα- NF-κB signaling. For example, atherosclerosis can induce the expression of Vcam-1, Icam-1, Cxcl12, and Cx3cl1 in SMCs, which in turn facilitates binding to monocytes [27]. TNFα can also induce cerebral SMC phenotypic changes through KLF4 and Myocardin, which may contribute to cerebral aneurysm formation [28]. In addition, many studies have demonstrated the enhancement of TNFα-NF-κB signaling in AAAs and the presence of modulated SMCs that express inflammatory markers [29]. NF-κB inhibition can slow the growth of aneurysms in animal models of AAA [30]. As a result, TNFα-NF-κB signaling might be a universal pathogenic response to injured vascular SMCs, and we are the first to demonstrate the ability of 17 β-estradiol to mitigate this response in Marfan aneurysms. We also found that 17 β-estradiol prevented the infiltration of adventitial inflammatory cell foci in the aortic root of Marfan mice. Recent studies using lineage tracing of aortic SMCs have shown that aortic injury can induce a phenotypic shift in SMCs to an inflammatory-like state, and these modified cells can migrate to the adventitia [31]. Future studies involving similar lineage tracing techniques will determine if some of the adventitial inflammatory cells in Marfan aortic roots originate from modified SMCs in the media that translocate to the adventitia or if modified SMCs in the media attract circulating inflammatory cells to invade the aortic wall or both. These future studies will also clarify if 17 β-estradiol acts only in the media and/or systemically to curtail the inflammatory response.

There are a few limitations to this study. Angiotensin II is used to accelerate the Marfan phenotype and increase the incidence of dissection/rupture as the *Fbn1^C1041G/+^* mouse rarely dissects. This strategy has been used by other groups in the past, and molecular pathway analysis of this provocation has been shown to resemble a severe Marfan phenotype [32]. An alternative approach would be to use the Fibrillin-1 hypomorphic mouse (mgR/mgR) model of Marfan aortopathy, and studies evaluating the effects of 17 β-estradiol in this model should be done in the future to corroborate the findings shown in this manuscript. Another limitation of the *Fbn1^C1041G/+^* mouse is the large spectrum of disease severity observed from mouse to mouse. This partially explains some of the variability seen in several molecular analyses throughout the paper, but we feel this recapitulates the variability seen in human Marfan patients. In addition, some studies have shown Vinculin levels to increase in some models of dissection [33]. We did not observe this finding in our aneurysm samples, and thus, we feel confident that it serves as an adequate loading control for protein abundance.

Moreover, this study was powered and designed to specifically describe the effects of estrogen in Male mice. The female mice are included for qualitative instead of quantitative comparison. Lastly, human aortic SMCs were derived from the aorta of failing recipient heart transplant patients. As such, residual pathology, such as increased arterial stiffness, may alter these cells relative to normal human cells. That being said, we examined the response to stimulation within each cell line, not a comparison to another control group. Marfan aorta also demonstrate increased tissue stiffness in humans and mice. Thus while the human data should be interpreted cautiously, it provides a useful example of human relevance for the molecular mechanisms under study here. Future studies will examine these pathways in other cell types like fibroblasts, endothelial cells, and immune cells to better understand how these cells use similar pathways to communicate and remodel tissue.

## 4. Materials and Methods

### 4.1. Mice

All animal procedures were approved by the Cedars-Sinai Medical Center Institution Animal Care and Use protocol. Male and female wild-type or *Fbn1^C1041G/+^* Marfan mice were maintained on a 129 genetic background with >20 back-crossings. 17 β-estradiol treatment was accomplished through anesthetizing 8-week-old mice with continuous isoflurane gas. The dorsum of mice was shaved and sterilized with betadine before making a sub-centimeter subcutaneous incision to place a 17 β-estradiol pellet (0.25 mg/pellet 60-day release, Innovative Research of America, Sarasota, FL). Sham surgery consisting of a similar dorsal incision that is open and closed was performed on littermate controls. Mice were imaged by transthoracic echocardiography to measure the size of the aortic root at the level of the sinus of Valsalva and the size of the ascending aorta. This was done every two weeks from the surgery date until animal sacrifice with isoflurane gas eight weeks later. For aortic rupture studies, 8-week-old mice were treated with 17 β-estradiol pellet or control surgery for four weeks, followed by implantation of subcutaneous mini-osmotic pumps (Azlet, Cupertino, CA, USA) for the continuous infusion of angiotensin II (1000 ng/kg/min). Surgical implantation of pumps was conducted in a similar matter described for pellet placement. Mice were aged for an additional one week for tissue harvesting and RNA expression analysis or four weeks for survival analysis (Supplemental Figure S1A). Aortic rupture was recorded at the time of autopsy by blood in the thoracic and/or abdominal cavity adjacent to the aorta. Root dissection was reported in mice with no evidence of rupture but with red blood cells in the aortic wall on a histological section through the sinus of Valsalva stained with H + E.

### 4.2. Histology

After animal sacrifice, mice were perfused with 10 cc of cold phosphate buffer saline through the left ventricle. The heart was transected across approximately the atrioventricular groove, and the ascending aorta, arch, and distal thoracic aorta were dissected from the mouse. The heart and aortic tissue were placed in 4% paraformaldehyde for 24 h at 4 °C and then transitioned to 70% ethanol. Fixed tissue was embedded in paraffin and sectioned at the aortic root for H + E, trichome, and elastin staining. Images were taken using the ECHO Revolve microscope (ECHO, San Diego, CA, USA). For elastin break quantification, four high power fields were captured from one 5 μm section across the aortic root at the level of the sinus of Valsalva. Elastin breaks were counted in each field within a 100 × 20 μm region of interest and averaged.

### 4.3. Western Blot

After animal sacrifice, aortic root tissue was dissected from the aortic valve to the sinotubular junction and immediately flash-frozen in liquid nitrogen. Protein lysates from RIPA buffer were reduced in loading buffer, run on acrylamide gels, and transferred to polyvinylidene fluoride membranes. The membranes were then probed with antibodies against Mmp2 and Mmp9 (Cell Signaling, Danvers, MA, USA) and Vinculin (Abcam, Waltham, MA, USA). Quantification was performed on ImageQuant^TM^ LAS4000 (GE) with the use of Vinculin as a loading control.

### 4.4. Cell Culture

Aortic smooth muscle cells were extracted from four male Marfan mice, and human aortic smooth muscle cells were extracted from four different non-aneurysmal aortas from heart transplant recipients. Protocols for the isolation and culture of human and murine smooth muscle cells have been previously published [34,35]. All procedures for the handling and procurement of human tissue were followed in accordance with the ethical standards of the UCLA School of Medicine institutional review board, and written consent was obtained from each patient. Cells were never split beyond eight passages. Cells were grown in phenol red-free DMEM/F-12 with 10% charcoal-stripped serum until 90% confluent. Cells were then serum starved and pre-treated with 17 β-estradiol (1 μM, Sigma, Waltham, MA, USA) or vehicle (equal volume of 0.05% ethanol in sterile water) for 24 h followed by 8 h of recombinant TNFα (2 ng/mL, R&D Systems, Minneapolis, MN, USA) or vehicle (equal volume of sterile water). Similar experiments were conducted with pre-treatment of cells for 2 h with pyrrolidine dithiocarbamate (PDTC, 50 μM, Sigma, Waltham, MA, USA) or vehicle followed by 8 h of TNFα (2 ng/mL) or vehicle.

### 4.5. RNA Isolation and Quantitative Real-Time PCR (qPCR)

Cells or aortic root tissue were harvested in TRIzol (Thermo Fischer, Waltham, MA, USA), and RNA was purified with phenol/chloroform. Reverse transcriptase (Applied Biosystems, Waltham, MA, USA) followed by quantitative real-time PCR (qPCR) was then performed with SYBR green (Bio-rad, Hercules, CA, USA). Ct values for RNA transcripts were normalized to *Gapdh* expression using the Delta-Delta Ct method. Statistical significance was determined using a Kruskal-Wallis test. The mouse primer sequences include: *Gapdh* f: GGCATTGCTCTCAATGACAA r: ATGTAGGCCATGAGGTCCAA, *C3* f: ACCTTACCTCGGCAAGTTTCT r: TTGTAGAGCTGCTGGTCAGG, *Mcp-1* f: GCATCCACGTGTTGGCTCA r: CTCCAGCCTACTCATTGGGATCA, *Vcam-1* f: TGAACCCAAACAGAGGCAGAGT r: GGTATCCCCATCACTTGAGCAGG, *Il-6* f: TGAACAACGATGATGCACTTG r: CTGAAGGACTCTGGCTTTGTC. The human sequences include: *GAPDH* f: ATGGGGAAGGTGAAGGTCG r: GGGGTCATTGATGGCAACAATA, *C3* f: ATCATCGGGAAGGACACTTG r: TTATCTGGAGTGGGGGAATG, *MCP-1* f: CCCCAGTCACCTGCTGTTAT r: AGATCTCCTTGGCCACAATG, VCAM-1 f: ATGGGGAAGGTGAAGGTCG r: CACAGGATTTTCGGAGCA, *Il-6* f: CACAGGATTTTCGGAGCA r: TGAGGTGCCCATGCTACATTT.

### 4.6. RNA-Seq

RNA from mouse aortic roots was extracted as described above. Libraries were prepped, and sequencing was performed on the Illumina HiSeq3000 (Illumina, San Diego, CA, USA). The sequencing depth was roughly around 30 million reads per sample. Raw reads were analyzed for quality using FASTqc version 0.11.9 [36]. Reads were aligned using TopHat2 version 2.1.1 [37], and reads were counted using HTseq version 2.0.2 [38]. Differential expression and principle component analysis were performed with DESeq2 [39] and pathway analysis was conducted with the gene set enrichment algorithm (GSEA) [40]. Transcription factor target enrichment was performed on the TRUUST database from the Enrichr bioinformatic platform [41]. Gene ontology was performed using ShinyGO version 0.77 [42]. Plots were made in R studio using ggplots2 version 3.4.0 [43]. Significant differentially expressed genes used the cutoff adjusted *p*-value < 0.1 and absolute fold change >1.5. The adjusted *p*-value for multiple comparisons uses the Benjamini Hochberg method per default settings of DESeq2.

### 4.7. Immunofluorescence

Paraffin-embedded tissue underwent deparaffinization in xylene and hydrated gradually from ethanol to water. Slides were then subjected to antigen retrieval in tris buffer at 95 °C for 30 min before blocking, primary antibody application overnight at 4 °C, and secondary antibody treatment for 1 h at room temperature. DAPI nuclear stain and immunostaining were conducted with antibodies against αSMA (Abcam, Fremont, CA, USA) and CD45 (Invitrogen, Waltham, MA, USA). Images were taken using the ECHO Revolve microscope (ECHO, San Diego, CA, USA) and quantified using ImageJ version 1.53 [44]. For each sample, one low-powered field was captured from one 5 μm section at the level of the sinus of Valsalva. The entire area encompassing the aortic root from intima to adventitia was encircled, and the mean fluorescent intensity was quantified.

### 4.8. Statistics

Aortic root size, ascending aorta size, mouse weight measurements, and in vitro RNA expression data are expressed as mean +/− standard error. Descriptive statistics for aortic size quotients include median and interquartile range (IQR). Kruskal-Wallis is used to describe statistical significance between groups in all studies. Survival significance was determined using the Wilcoxon method. A power analysis to determine the number of mice required to detect a difference in aortic root size was conducted assuming an aortic root size standard deviation of 0.25 mm at 16 weeks of age, a mean aortic root size of 3 mm, and a predicted effect reduction of 10% in the presence of 17 β-estradiol. This yielded a total sample size of 22 mice for 80% power and a *p*-value of 0.05. Graphs and statistical analysis were performed in JMP version 16 (Cary, NC, USA) or R studio version 4.2.2 [45].

## 5. Conclusions

In summary, we describe the effects of 17 β-estradiol on the reduction of aortic root aneurysms and the incidence of aortic rupture in a Marfan mouse model. Mechanistically, 17 β-estradiol appears to quell inflammation by blocking TNFα-NF-κB mediated induction of pro-inflammatory genes. These results provide several novel potential targets that could be used therapeutically to treat aortic aneurysms and dissection.

## Data Availability

RNA-seq data will be uploaded upon acceptance to the Gene Expression Omnibus.

## Supplementary Materials

The following supporting information can be downloaded at https://www.mdpi.com/article/10.3390/ijms241713571/s1.

## References

[1] Hiratzka L.F., Bakris G.L., Beckman J.A., Bersin R.M., Carr V.F., Casey D.E., Eagle K.A., Hermann L.K., Isselbacher E.M., Kazerooni E.A. (2010). 2010 ACCF/AHA/AATS/ACR/ASA/SCA/SCAI/SIR/STS/SVM Guidelines for the diagnosis and management of patients with thoracic aortic disease. A Report of the American College of Cardiology Foundation/American Heart Association Task Force on Practice Guidelines, American Association for Thoracic Surgery, American College of Radiology, American Stroke Association, Society of Cardiovascular Anesthesiologists, Society for Cardiovascular Angiography and Interventions, Society of Interventional Radiology, Society of Thoracic Surgeons, and Society for Vascular Medicine. J. Am. Coll. Cardiol..

[2] Cheung K., Boodhwani M., Chan K.-L., Beauchesne L., Dick A., Coutinho T. (2017). Thoracic Aortic Aneurysm Growth: Role of Sex and Aneurysm Etiology. J. Am. Heart Assoc..

[3] Grubb K.J., Kron I.L. (2011). Sex and gender in thoracic aortic aneurysms and dissection. Semin. Thorac. Cardiovasc. Surg..

[4] Ailawadi G., Eliason J.L., Roelofs K.J., Sinha I., Hannawa K.K., Kaldjian E.P., Lu G., Henke P.K., Stanley J.C., Weiss S.J. (2004). Gender differences in experimental aortic aneurysm formation. Arterioscler. Thromb. Vasc. Biol..

[5] Martin-McNulty B., Tham D.M., Cunha V.d., Ho J.J., Wilson D.W., Rutledge J.C., Deng G.G., Vergona R., Sullivan M.E., Wang Y.-X. (2003). B-Estradiol Attenuates Development of Angiotensin II Induced Aortic Abdominal Aneurysm in Apolipoprotein E Deficient Mice. Arterioscler. Thromb. Vasc. Biol..

[6] Chen X., Rateri D.L., Howatt D.A., Balakrishnan A., Moorleghen J.J., Cassis L.A., Daugherty A. (2016). TGF-β Neutralization Enhances AngII-Induced Aortic Rupture and Aneurysm in Both Thoracic and Abdominal Regions. PLoS ONE.

[7] Groth K.A., Stochholm K., Hove H., Kyhl K., Gregersen P.A., Vejlstrup N., Østergaard J.R., Gravholt C.H., Andersen N.H. (2017). Aortic events in a nationwide Marfan syndrome cohort. Clin. Res. Cardiol..

[8] Gharraee N., Sun Y., Swisher J.A., Lessner S.M. (2022). Age and sex dependency of thoracic aortopathy in a mouse model of Marfan syndrome. Am. J. Physiol. Heart Circ. Physiol..

[9] Jiménez-Altayó F., Siegert A.M., Bonorino F., Meirelles T., Barberà L., Dantas A.P., Vila E., Egea G. (2017). Differences in the Thoracic Aorta by Region and Sex in a Murine Model of Marfan Syndrome. Front. Physiol..

[10] Albornoz G., Coady M.A., Roberts M., Davies R.R., Tranquilli M., Rizzo J.A., Elefteriades J.A. (2006). Familial thoracic aortic aneurysms and dissections--incidence, modes of inheritance, and phenotypic patterns. Ann. Thorac. Surg..

[11] Tashima Y., He H., Cui J.Z., Pedroza A.J., Nakamura K., Yokoyama N., Iosef C., Burdon G., Koyano T., Yamaguchi A. (2020). Androgens Accentuate TGF-β Dependent Erk/Smad Activation During Thoracic Aortic Aneurysm Formation in Marfan Syndrome Male Mice. J. Am. Heart Assoc..

[12] Hsieh Y.C., Yang S., Choudhry M.A., Yu H.P., Bland K.I., Schwacha M.G., Chaudry I.H. (2006). Flutamide restores cardiac function after trauma-hemorrhage via an estrogen-dependent pathway through upregulation of PGC-1. Am. J. Physiology. Heart Circ. Physiol..

[13] Metzger D.L., Kerrigan J.R. (1993). Androgen receptor blockade with flutamide enhances growth hormone secretion in late pubertal males: Evidence for independent actions of estrogen and androgen. J. Clin. Endocrinol. Metab..

[14] Boese A.C., Kim S.C., Yin K.J., Lee J.P., Hamblin M.H. (2017). Sex differences in vascular physiology and pathophysiology: Estrogen and androgen signaling in health and disease. Am. J. Physiol. Heart Circ. Physiol..

[15] Xing D., Nozell S., Chen Y.-F., Hage F., Oparil S. (2009). Estrogen and Mechanisms of Vascular Protection. Arterioscler. Thromb. Vasc. Biol..

[16] Shimizu K., Mitchell R.N., Libby P. (2006). Inflammation and Cellular Immune Responses in Abdominal Aortic Aneurysms. Arterioscler. Thromb. Vasc. Biol..

[17] He R., Guo D.C., Sun W., Papke C.L., Duraisamy S., Estrera A.L., Safi H.J., Ahn C., Buja L.M., Arnett F.C. (2008). Characterization of the inflammatory cells in ascending thoracic aortic aneurysms in patients with Marfan syndrome, familial thoracic aortic aneurysms, and sporadic aneurysms. J. Thorac. Cardiovasc. Surg..

[18] Guo G., Booms P., Halushka M., Dietz H.C., Ney A., Stricker S., Hecht J., Mundlos S., Robinson P.N. (2006). Induction of Macrophage Chemotaxis by Aortic Extracts of the mgR Marfan Mouse Model and a GxxPG-Containing Fibrillin-1 Fragment. Circulation.

[19] Cavanaugh N.B., Qian L., Westergaard N.M., Kutschke W.J., Born E.J., Turek J.W. (2017). A Novel Murine Model of Marfan Syndrome Accelerates Aortopathy and Cardiomyopathy. Ann. Thorac. Surg..

[20] Xing D., Feng W., Miller A.P., Weathington N.M., Chen Y.F., Novak L., Blalock J.E., Oparil S. (2007). Estrogen modulates TNF-alpha-induced inflammatory responses in rat aortic smooth muscle cells through estrogen receptor-beta activation. Am. J. Physiol. Heart Circ. Physiol..

[21] Villard C., Eriksson P., Kronqvist M., Lengquist M., Jorns C., Hartman J., Roy J., Hultgren R. (2017). Differential expression of sex hormone receptors in abdominal aortic aneurysms. Maturitas.

[22] Sokolis D.P., Iliopoulos D.C. (2014). Impaired mechanics and matrix metalloproteinases/inhibitors expression in female ascending thoracic aortic aneurysms. J. Mech. Behav. Biomed. Mater..

[23] Xing D., Oparil S., Yu H., Gong K., Feng W., Black J., Chen Y.F., Nozell S. (2012). Estrogen modulates NFκB signaling by enhancing IκBα levels and blocking p65 binding at the promoters of inflammatory genes via estrogen receptor-β. PLoS ONE.

[24] Ghisletti S., Meda C., Maggi A., Vegeto E. (2005). 17beta-estradiol inhibits inflammatory gene expression by controlling NF-kappaB intracellular localization. Mol. Cell Biol..

[25] Belguise K., Sonenshein G.E. (2007). PKCtheta promotes c-Rel-driven mammary tumorigenesis in mice and humans by repressing estrogen receptor alpha synthesis. J. Clin. Investig..

[26] Nakamura K., Dalal A.R., Yokoyama N., Pedroza A.J., Kusadokoro S., Mitchel O., Gilles C., Masoudian B., Leipzig M., Casey K.M. (2023). Lineage-Specific Induced Pluripotent Stem Cell-Derived Smooth Muscle Cell Modeling Predicts Integrin Alpha-V Antagonism Reduces Aortic Root Aneurysm Formation in Marfan Syndrome Mice. Arterioscler. Thromb. Vasc. Biol..

[27] Alexander M.R., Murgai M., Moehle C.W., Owens G.K. (2012). Interleukin-1β modulates smooth muscle cell phenotype to a distinct inflammatory state relative to PDGF-DD via NF-κB-dependent mechanisms. Physiol. Genom..

[28] Ali M.S., Starke R.M., Jabbour P.M., Tjoumakaris S.I., Gonzalez L.F., Rosenwasser R.H., Owens G.K., Koch W.J., Greig N.H., Dumont A.S. (2013). TNF-α induces phenotypic modulation in cerebral vascular smooth muscle cells: Implications for cerebral aneurysm pathology. J. Cereb. Blood Flow Metab..

[29] Cao G., Xuan X., Hu J., Zhang R., Jin H., Dong H. (2022). How vascular smooth muscle cell phenotype switching contributes to vascular disease. Cell Commun. Signal..

[30] Parodi F.E., Mao D., Ennis T.L., Bartoli M.A., Thompson R.W. (2005). Suppression of experimental abdominal aortic aneurysms in mice by treatment with pyrrolidine dithiocarbamate, an antioxidant inhibitor of nuclear factor-kappaB. J. Vasc. Surg..

[31] Majesky M.W., Horita H., Ostriker A., Lu S., Regan J.N., Bagchi A., Dong X.R., Poczobutt J., Nemenoff R.A., Weiser-Evans M.C. (2017). Differentiated Smooth Muscle Cells Generate a Subpopulation of Resident Vascular Progenitor Cells in the Adventitia Regulated by Klf4. Circ. Res..

[32] Gensicke N.M., Cavanaugh N.B., Andersen N.D., Huang T., Qian L., Dyle M.C., Turek J.W. (2020). Accelerated Marfan syndrome model recapitulates established signaling pathways. J. Thorac. Cardiovasc. Surg..

[33] Wang H.Q., Yang H., Tang Q., Gong Y.C., Fu Y.H., Wan F., Yang B., Guo R., Zhong Y.L., Zhu J.M. (2020). Identification of Vinculin as a Potential Diagnostic Biomarker for Acute Aortic Dissection Using Label-Free Proteomics. BioMed Res. Int..

[34] MacFarlane E.G., Parker S.J., Shin J.Y., Kang B.E., Ziegler S.G., Creamer T.J., Bagirzadeh R., Bedja D., Chen Y., Calderon J.F. (2019). Lineage-specific events underlie aortic root aneurysm pathogenesis in Loeys-Dietz syndrome. J. Clin. Investig..

[35] Lu S., Sun X., Hong T., Song K., Yang S., Wang C. (2013). Isolation and culture of smooth muscle cells from human acute type A aortic dissection. J. Cardiothorac. Surg..

[36] FastQC: A Quality Control Tool for High Throughput Sequence Data. http://www.bioinformatics.babraham.ac.uk/projects/fastqc.

[37] Kim D., Pertea G., Trapnell C., Pimentel H., Kelley R., Salzberg S.L. (2013). TopHat2: Accurate alignment of transcriptomes in the presence of insertions, deletions and gene fusions. Genome Biol..

[38] Putri G.H., Anders S., Pyl P.T., Pimanda J.E., Zanini F. (2022). Analysing high-throughput sequencing data in Python with HTSeq 2.0. Bioinformatics.

[39] Love M.I., Huber W., Anders S. (2014). Moderated estimation of fold change and dispersion for RNA-seq data with DESeq2. Genome Biol..

[40] Subramanian A., Tamayo P., Mootha V.K., Mukherjee S., Ebert B.L., Gillette M.A., Paulovich A., Pomeroy S.L., Golub T.R., Lander E.S. (2005). Gene set enrichment analysis: A knowledge-based approach for interpreting genome-wide expression profiles. Proc. Natl. Acad. Sci. USA.

[41] Chen E.Y., Tan C.M., Kou Y., Duan Q., Wang Z., Meirelles G.V., Clark N.R., Ma’ayan A. (2013). Enrichr: Interactive and collaborative HTML5 gene list enrichment analysis tool. BMC Bioinform..

[42] Ge S.X., Jung D., Yao R. (2019). ShinyGO: A graphical gene-set enrichment tool for animals and plants. Bioinformatics.

[43] Wickham H. (2016). ggplot2: Elegant Graphics for Data Analysis.

[44] Schneider C.A., Rasband W.S., Eliceiri K.W. (2012). NIH Image to ImageJ: 25 years of image analysis. Nat. Methods.

[45] RStudio Team (2019). RStudio: Integrated Development for R.

