# Supplementary material for: 17 β-Estradiol Impedes Aortic Root Dilation and Rupture in Male Marfan Mice
Source: Int J Mol Sci. 2023 Sep 1;24(17):13571. doi: 10.3390/ijms241713571 (PMC10487461; doi:10.3390/ijms241713571)

Supplemental Figures:

Figure S1

17 β-estradiol does not alter ascending aorta growth, weight, or aortic root elastin breaks. A.) Experimental design of drugs administered and duration of treatment. B.) Ascending aorta size and weight C.) of 8 week old mice treated with 17 β-estradiol or sham surgery measured every 2 weeks for 8 weeks. D.) Quantification of elastin breaks in wild type, Marfan, and Marfan plus 17 β-estradiol male mice. Data are mean +/- standard error. E2: 17 β-estradiol, MFS: Marfan syndrome, Sham: sham surgery, WT: Wild-type.

Figure S2

Marfan mice have reduced expression of contractile proteins. Normalized read counts of A. the estrogen response gene *Greb1*, B. TNFα, and C. contractile proteins *aSMA, Tagln, Cnn1, and Myh11* from RNAseq performed on aortic root tissue of male wild-type (n=5), Marfan (n=5), Marfan treated with 8 weeks of 17 β-estradiol (n=5), and 7 days of angiotensin II treated-wild type (n=4), Marfan (n=4), and Marfan mice treated with 5 weeks of 17 β-estradiol (n=4). Box plots describe quartiles. Adjusted p-values from DESeq2 for pairwise comparisons are depicted above charts. AII: angiotensin II, MFS: Marfan syndrome, E2: 17 β-estradiol.

Figure S3

17 β-estradiol mitigates TNFα medicated NF-κB gene expression in human aortic SMCs. A.) Expression of *MCP-1, VCAM-1, IL-6, and C3* in n=4 independent male human aortic SMC lines pre-treated with 17 β-estradiol or vehicle followed by the addition of TNFα or vehicle. Bar charts display mean +/- standard error. Relative expression is normalized to the cytokine treated group. E2: 17 β-estradiol.

Supplemental Figure S1

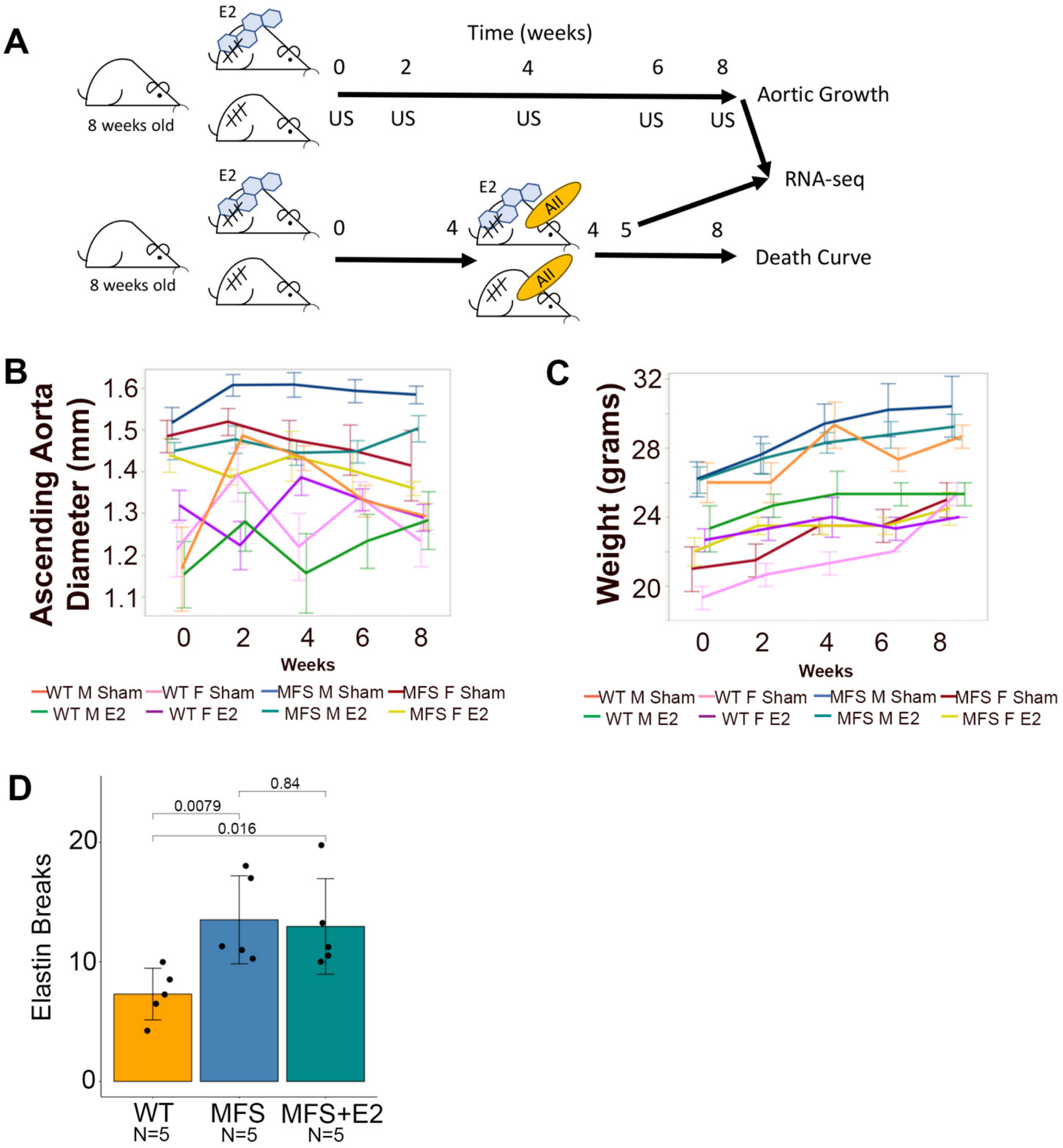

Supplemental Figure S2

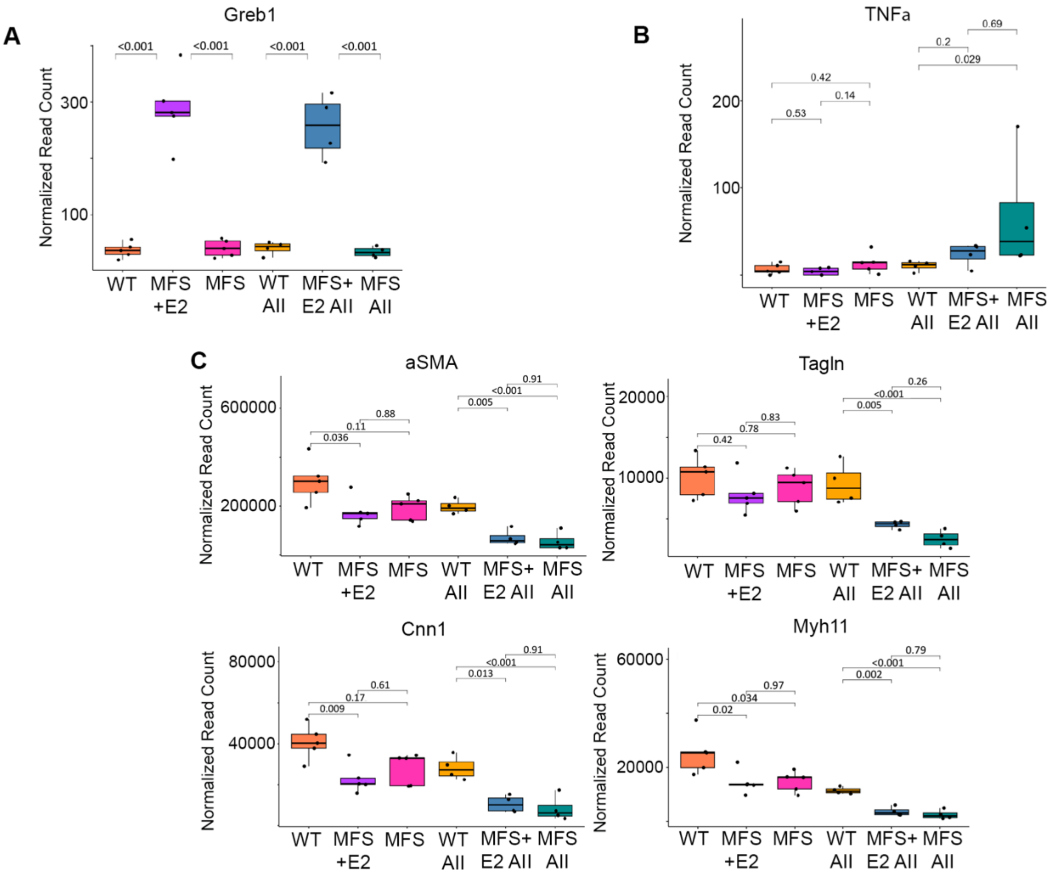

Supplemental Figure S3

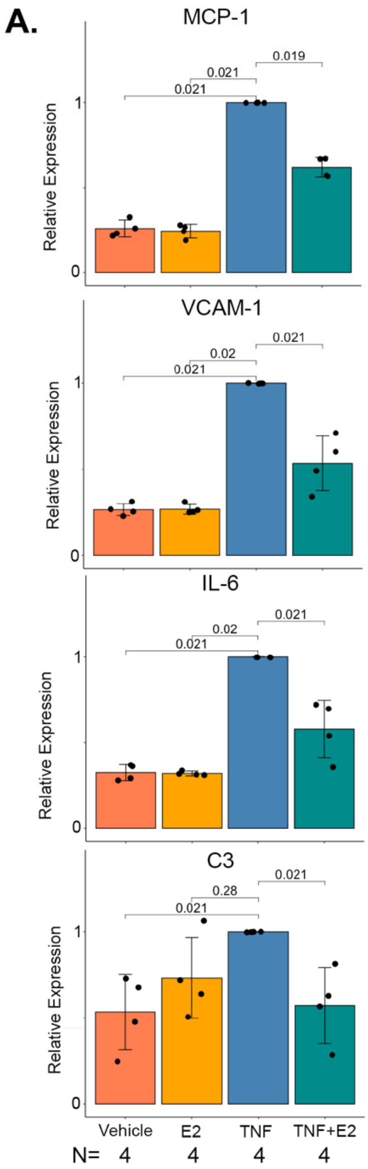

Supplement: Supplementary file 1 [file ijms-24-13571-s001.zip › ijms-2539438-supplementary.pdf]
